# Analysis of Phenolic Compounds in Buckwheat (*Fagopyrum esculentum* Moench) Sprouts Modified with Probiotic Yeast

**DOI:** 10.3390/molecules27227773

**Published:** 2022-11-11

**Authors:** Marta Molska, Julita Reguła, Ireneusz Kapusta, Michał Świeca

**Affiliations:** 1 Department of Human Nutrition and Dietetics, Faculty of Food Sciences and Nutrition, Poznan University of Life Sciences, 28 Wojska Polskiego Street, 60-637 Poznan, Poland; 2 Department of Food Technology and Human Nutrition, Institute of Food Technology and Nutrition, University of Rzeszow, Zelwerowicza 4 St., 35-601 Rzeszow, Poland; 3 Department of Food Chemistry and Biochemistry, University of Life Sciences in Lublin, Skromna Str. 8, 20-704 Lublin, Poland

**Keywords:** phenolic compounds, antioxidant activity, anti-inflammatory activity, dietary fiber, nutraceutical potential, germination, common buckwheat, *Saccharomyces boulardii*

## Abstract

Buckwheat sprouts are a source of various nutrients, e.g., antioxidant flavonoids, which have a positive effect on human health. This study analyzed the content of phenolic compounds and assessed their impact on the antioxidant and anti-inflammatory properties and dietary fiber in modified buckwheat sprouts. For this purpose, the buckwheat seeds were modified by adding *Saccharomyces cerevisiae* var. *boulardii*. The modified buckwheat sprouts showed a higher content of total phenol compounds (1526 µg/g d.w.) than the control sprouts (951 µg/g d.w.) and seeds (672 µg/g d.w.). As a consequence, a higher antioxidant activity and anti-inflammatory effect were noted. Probiotic-rich sprouts also had the highest content of total dietary fiber and its soluble fraction. A correlation between phenolic compounds and the antioxidant and anti-inflammatory effects, as well as dietary fiber, was shown. The interaction between dietary fiber and phenolic compounds affects the bioaccessibility, bioavailability, and bioactivity of phenolic compounds in food. The introduction of probiotic yeast into the sprouts had a positive effect on increasing their nutritional value, as well as their antioxidant and anti-inflammatory activity. As a consequence, the nutraceutical potential of the raw material changed, opening a new direction for the use of buckwheat sprouts, e.g., in industry.

## 1. Introduction

Buckwheat belongs to the *Polygonaceae* family and is classified as a pseudocereal. Common buckwheat (*Fagopyrum esculentum* Moench) and Tartary buckwheat (*Fagopyrum tataricum* (L.) Gaertn.) are the main species of buckwheat consumed by humans [1,2].

Buckwheat (*Fagopyrum esculentum* Moench) is a crop mainly grown for its seeds [3]. There are various buckwheat-based foods on the market today, such as bread, pasta, tea, sprouts, and vinegar [4]. Sprouts are recognized as a unique dietary vegetable in Asia, Europe, and the United States. They are gaining more and more attention, as consumers require minimally processed, no additive, natural, nutritious, and wholesome food. It is worth paying attention to the germination process; for example, buckwheat sprouts’ total phenolics and antioxidant capacity are greater than that of seeds [5].

Food of plant origin is receiving increasing interest, in terms of its protective effect against various diseases; in addition to its main role, that is, providing essential nutrients [6,7]. Buckwheat is a source of many antioxidants such as polyphenols, including six main flavonoids: rutin, orientin, vitexin, quercetin, isovitexin, and isoorientin [1,8]. Polyphenols are plant components that influence many chemical and biochemical reactions taking place in plant tissue. As components of plant raw materials used in nutrition, they influence sensory values and biological activity. Therefore, polyphenols are responsible for taste and color, as well as for antioxidant activity and health-promoting effects [9,10,11].

Abiotic and biotic factors affect the quality and quantity of crops. Quality can be defined as agronomic (e.g., fruit size, yield, and resistance to bacteria and fungi), organoleptic (e.g., color, firmness, and shape), and the nutrient and vitamin content. Stress from unfavorable stimuli can reduce yields. Abiotic factors include the soil composition, acidity, extreme salinity, high and low temperatures, drought, pollution, rain, wind, humidity, and ultraviolet radiation. On the other hand, biotic factors include various fungi, bacteria, and viruses that can cause many diseases [4,12,13].

Plants try to defend themselves and adapt to ecosystem conditions by releasing oxygen through photosynthesis, metabolic processes such as respiration, and the regulation of reactive oxygen species (ROS) [14]. Oxidative stress is described as an imbalance between the elimination of free radicals (oxidants) and production. The properties of antioxidants have been revealed, and they are considered protective against cancer and cardiovascular disease [15]. Buckwheat sprouts are rich in flavonoid compounds, thanks to which they are considered a health-promoting product with antioxidant and antihypertensive properties [1,16].

Germination is a process in which the primary, as well as secondary, active metabolites are increased. This is due to the fact that the seeds undergo a series of morphological and physiological changes. Active phytochemicals (e.g., phenols and flavonoids) gradually increase during the germination process. A higher level of polyphenolic compounds translates into the higher antioxidant activity of buckwheat sprouts. It is worth noting that different flavonoids may contribute differently to the overall antioxidant activity of different buckwheat species [17,18].

Flavonoids may be a promising molecule for solving chronic inflammatory processes. The results obtained in the publication by Ribeiro et al. 2015 showed promising effect of flavonoids in modulating the inflammatory process (namely those that represent a catechol group in the B-ring). This is because some flavonoids were able to simultaneously inhibit the production of pro-inflammatory prostaglandin E2 and pro-inflammatory cytokines, due to their ability to scavenge free radicals. In this way, they are excellent candidates for the prevention of diseases associated with increased production of free radicals, including cancer [14,19]. Buckwheat flavonoids were able to induce apoptosis in human leukemic cells HL-60 [20]. Ishii et al. [10] reported the anti-inflammatory effects of buckwheat sprouts on human colon cancer cells [21].

Buckwheat sprouts showed a strong anti-inflammatory effect by inhibiting inflammatory cytokines (e.g., IL-6), some inflammatory mediators (e.g., COX-2), and tumor necrosis factor-α (TNF-α). The anti-inflammatory properties of buckwheat sprouts are attributed to their rich content of phenolic compounds [4].

Taking into account the above information, it is necessary to develop effective techniques for the effective recovery/extraction and accurate determination of phenolic compounds. Phenolic compounds in plants are usually bound or free. The bound phenolic compounds are usually isolated by acid and alkali hydrolysis, followed by solvent extraction. Whereas, free phenolic compounds are usually extracted from plant samples using organic solvents (e.g., methanol, ethanol, and acetone) [22,23].

Probiotics are “live strains of strictly selected microorganisms which, when administered in adequate amounts, confer a health benefit on the host”. They can affect food’s organoleptic and microbiological quality, thanks to which they are used in traditional food products, e.g., fermented milk products. Moreover, probiotics can be used as part of products, which are their carriers. In recent years, e.g., chocolate and fruit drinks have been used as the probiotic carriers [24,25].

Another possibility of using probiotic microorganisms is to modify raw materials by adding them. Sprouts’ metabolism can be additionally modified to obtain a more intensive growth or an increased concentration of bioactive ingredients. On the other hand, in the study by Świeca et al., 2019, the authors showed that legume sprouts enriched with *Saccharomyces cerevisiae* var. *boulardii* represented a new functional product characterized by increased health and nutritional properties [26,27]. At the same time, buckwheat is gaining attention as a potential functional food. It is noted that both the raw material itself and products enriched with buckwheat are associated with many health benefits [28,29,30].

The germination process significantly increases the nutritional value by increasing the bioavailability of certain nutrients, e.g., vitamins [31]. Modifying the seeds by adding probiotics may also change the bioavailability of the ingredients, as well as their quantity. In the publication by Molska et al., in 2022, it was noticed that the protein availability was lower in the probiotic group. However, this was compensated for by the higher amount of protein in this group [32]. In subsequent studies, changes were noticed in the composition and amounts of individual sterols, stanols, and fatty acids [29,30]. Moreover, it has been also proven that co-culture of sprouts and bacteria affects the microbiological quality of the final product, slightly decreasing the total count of mesophilic bacteria. After 3 days of sprouting, a single edible portion of sprouts enriched with probiotic yeast contained an amount classifying them as a probiotic product (6.5 log/100 g f.m.) [29].

Hence, in this study, modified buckwheat sprouts were analyzed. Production of buckwheat sprouts was modified by introducing the probiotic yeast *Saccharomyces cerevisiae* var. *boulardii* during the soaking process. Therefore, the hypotheses assumed that such a modification would change the content of bioactive compounds (phenolics, dietary fiber) and affect the nutraceutical potential (antioxidant and anti-inflammatory properties) of the obtained buckwheat sprouts. The aim of the research was to analyze the content of phenolic compounds and to evaluate their influence on the antioxidant and anti-inflammatory properties, as well as the dietary fiber in modified buckwheat sprouts.

## 2. Results and Discussion

### 2.1. Phenolic Compounds

**Figure 1:**
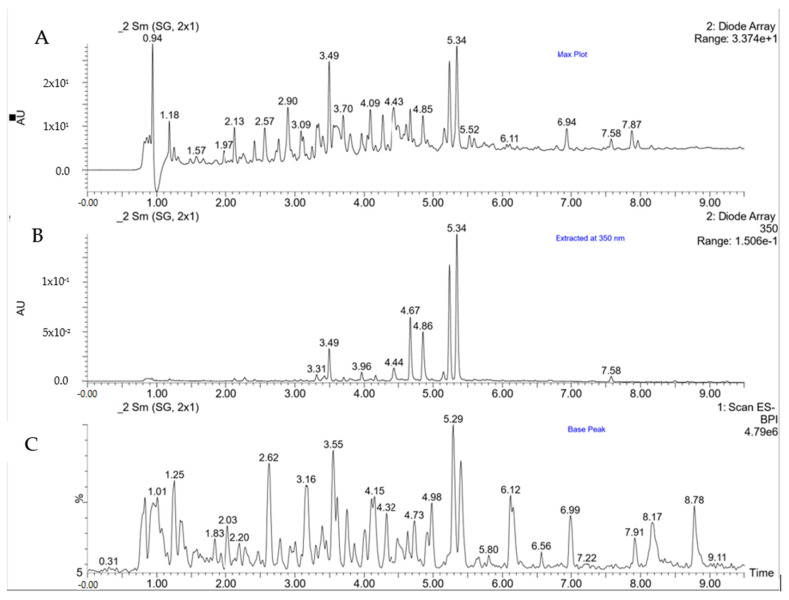
Chromatogram of buckwheat polyphenols obtained using UPLC-PDA-MS. (**A**)—Chromatogram UV-Vis recorded at max plot, (**B**)—chromatogram UV-VIS extracted at λ = 350 nm, (**C**)—base peak chromatogram recorded at full scan (ESI-MS).

In recent years, many studies have been carried out on the composition of polyphenols in food, as well as their bioavailability and metabolism. This increased interest in polyphenols may be due to the results of epidemiological studies that have linked the consumption of diets rich in plant foods with a reduced risk of diseases related to oxidative stress [33,34]. Table 1 presents the phenolic compounds identified in *Fagopyrum esculentum* Moench buckwheat seeds and sprouts. Figure 1 shows a chromatogram of buckwheat polyphenols obtained using the UPLC-PDA-MS method.

Three phenolic acids (caffeoyl-glucoside, caffeoyl-rhamnopyranosyl-glucopyranosyl-glucopyranoside, and caffeoyl-rhamnopyranosyl-glucopyranosyl) were identified in both the seeds and the sprouts of buckwheat. Caffeoyl-rhamnopyranosyl-glucopyranosyl was the dominant phenolic acid in the grains and buckwheat sprouts; their amount in seeds was 79.80 ± 0.30 µg/g d.w., control sprouts 92.04 ± 0.05 µg/g d.w., and probiotic-rich sprouts 118.23 ± 0.04 µg/g d.w. The caffeoyl content was 47.29 ± 0.41 µg/g d.w. in seeds, 53.23 ± 0.06 µg/g d.w. in control sprouts, and 55.48 ± 0.52 µg/g d.w. in probiotic-rich sprouts.

Seventeen flavan-3-ols have also been identified in common buckwheat. There were statistically significant differences between the three identified samples in the case of twelve compounds (unknown catechin derivate, (epi) afzelechin-(epi)-catechin, catechin-glucoside, catechin-3-O-glucoside-6-O-rutinoside, caffeoyl-glucoside, (+)catechin, catechin-glucoside, epicatechin-(4-8)-epigallocatechin-gallate, epicatechin gallate dimethyl derivative (-)epicatechin, catechin trimer, and epicatechin gallate methyl derivative). In the next five, there were statistically significant differences between the control sprouts and the probiotic-rich sprouts: epicatechin-(4-8)-epicatechin, epicatechin gallate, epicatechin trimer, epiafzelechin-epicatechin-gallate dimethyl derivative, and epiafzelechin-epicatechin-gallate methyl derivative.

Among all identified compounds, the modified buckwheat sprouts were characterized by the highest content of epicatechin gallate methyl derivative, i.e., 138.25 ± 0.98 µg/g d.w.; then, respectively, control sprouts (113.27 ± 0.02 µg/g d.w.) and seeds (7.20 ± 0.86 µg/g d.w.). This example shows that the germination process had a significant impact on the change in the content of bioactive compounds in the raw material. During the germination process, active phytochemicals such as phenols and flavonoids gradually increased in the buckwheat species [18].

It is noteworthy that the probiotic-rich sprouts also had the highest content of epicatechin-(4-8)-epicatechin and catechin-glucoside compared to the seeds and control sprouts. On the other hand, the lowest amount was found for the epiafzelechin-epicatechin-gallate methyl derivative, which was 12.32 ± 0.62 µg/g d.w. in the modified sprouts, 5.68 ± 0.16 µg/g d.w. in the control sprouts and 21.39 ± 0.2 µg/g d.w. in seeds. Comparing the content of flavan-3-ols in the control sprouts and sprouts rich in probiotics, it can be seen that the modification increased the amount of these bioactive compounds.

Regarding the flavanols, orientin, isoorientin, quercetin-3 O-rutinoside (rutin), and vitexin were identified. Orientin and vitexin were not identified in seeds. The flavanol with the highest amount in the raw material was vitexin, which increased by 100.94 µg/g d.w. compared to the control sprouts. In the publication of Wiczkowski et al. 2014, the authors found that the main flavonols in buckwheat were orientin, isoorietin, and vitexin, as well as isovitexin. On the other hand, in Tartary buckwheat sprouts, these were detected in negligible amounts or not at all [35,36].

Buckwheat grains, as well as sprouts, are important sources of rutin, and the content depends on the growing conditions and type [37]. However, it has been noticed that *Fagopyrum esculentum* Moench has a several-times-lower rutin content than *Fagopyrum tataricum* L. Gaertn. [38]. Rutin lowers high blood pressure and has antioxidant and lipid peroxidative effects. It is worth noting that it also has a lipid-lowering effect, reducing the absorption of cholesterol from the diet. It causes a lower cholesterol level in the liver and plasma [37,39].

**Figure 2:**
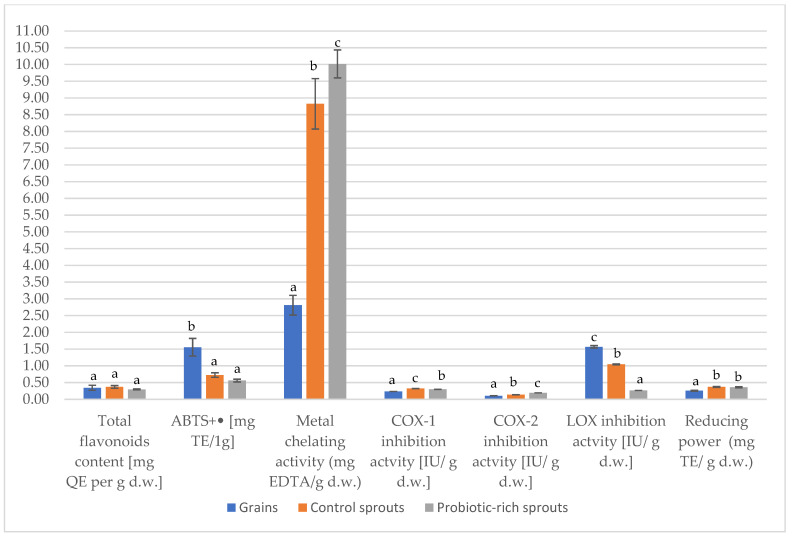
Comparison of the gastrointestinal digestibility index (IA). Mean values with different letters above the bars are statistically significantly different (*p* ≤ 0.05). Statistical analysis for each presented parameter was carried out separately.

The level and potential bioavailability of pro-health properties are presented in Table 2 and Figure 2. The majority of phenolics belong to the flavonoids. In this section, we focused only on the quantitative analysis of this group of metabolites. The total content of flavonoids decreased in the following order: probiotic-rich sprouts > control sprouts > grains (statistically significant values). In the publication of Qin et al. 2010, the authors showed that the total content of flavonoids in buckwheat seeds was 0.67–2.25 mg/g d.w. [40]. It can be seen that, in this case, the amount of flavonoids was more than two-times higher in the modified sprouts than in the control sprouts. However, in the presented study, the total content of flavonoids was 14.1 ± 0.64 (mg QE per g d.w.). The value after digestion was 4.88 ± 1.18 (mg QE per g d.w.). A similar situation was seen in the case of the sprouts.

It is worth noting that *Saccharomyces boulardii* influences the synthesis of active phytochemicals such as phenols, including isoflavones, and thereby increasing the antioxidant capacity of the products [41,42]. It is supposed that the increase in phenolics in co-culture may be due to different mechanisms. Previously it was proven that the components of yeast cell walls (chitin, chitosan) can act as effective elicitors promoting the de novo synthesis of pathogen-related compounds e.g., phenolics [43]. This strategy successfully increased the phenolic content and resulted from the pro-health properties of kidney bean [44] and wheat sprouts [45]. On the other hand, yeast effectively growing into the seed may cause a loosening of the seed structure, which promotes the release of bound phenolics from buckwheat cell walls [46].

### 2.2. Antioxidant Activity

The content of flavonoids in probiotic-rich sprouts was significantly higher than in the other forms of buckwheat. Consequently, the modified sprouts showed the highest antioxidant potential, expressed as, inter alia, the ability to quench cationic radical ABTS^+•^. A similar relationship was noticed in the publication by Kędzierska-Matysek et al. 2021, in the case of buckwheat honey [47].

It is noted that, simultaneously with the increase in the total content of flavonoids in sprouts, the reduction power and the ability to scavenge free radicals increased proportionally. ABTS^+•^ values ranged from 5.77 to 19.78 mg TE/g (before digestion). Of which the most active were the modified sprouts. In contrast, the seeds (11.24 mg TE/g) had the lowest reduction power, while the modified sprouts (31.15 mg TE/g) were the highest. The “bioavailability” of the reduction force decreased in the following direction: control sprouts > probiotic-rich sprouts > seeds.

The presence of transition metal ions plays a significant role in oxidative processes leading to the formation of superoxide anion radicals and singlet oxygen during the Fenton reaction and during changes during the storage and thermal processing of food. In addition, metal ions are catalysts for the decomposition of lipid hydroperoxides. These processes can be delayed by the chelation and deactivation of iron ions present in the system [48]. Flavonoids can indirectly chelate transition metal ions, i.e., copper and iron, which prevents the formation of reactive hydroxyl radicals in cells. They also contribute to the stabilization of cell membranes [49]. The chelating capacity of metals changed similarly to the indices mentioned above. However, what distinguishes this was the increase in the activity in the digestive process. After digestion, the values were higher: 35.39 ± 1.23 mg EDTA/g d.w. in seeds, 102.15 ± 8.10 mg EDTA/g d.w. in control sprouts, and 117.78 ± 4.95 mg EDTA/g d.w. in modified sprouts. The bioavailability of the chelating capacity was highest in the probiotic-rich sprouts (Figure 2).

The strong metal chelating activity of probiotic-rich sprouts could have been due to the higher content of quercetin compared to the seeds. It has more structural features than complex metal ions. The therapeutic benefits of chelators are noted in metal catalyzed chronic diseases induced by oxidative stress, e.g., in cardiovascular diseases [50,51]. The strong chelating activity of buckwheat metals may be due to the higher content of, not only quercitin, but also rutin, as they have more of the structural features of complexing metal ions [4].

### 2.3. Anti-Inflammatory Activity

The inhibition value of cycloxygenase 1 (COX-1) and 2 (COX-2) decreased analogously to the flavonoids, from probiotic-rich sprouts to seeds. The bioavailability of COX-1 was lowest in seeds and highest in control sprouts (Figure 2). In the case of COX-2, the bioavailability was lower, as follows: 0.11 in seeds, 0.14 in control sprouts, and 0.19 in probiotic-rich sprouts. COX-2 overexpression plays a key role in several inflammatory diseases. In contrast, inhibition of COX-2 expression is one of the therapeutic targets for inflammation [52]. The value of lipoxygenase inhibition in the modified vines was six times higher than in the probiotic-rich sprouts. The interest in lipoxygenases on the part of food technologists results from their ability to produce free radicals and peroxides, which are involved in the oxidation of vitamins, dyes, phenolic compounds, and proteins [53]. The anti-inflammatory properties of buckwheat sprouts have been attributed to their high content of phenolic compounds, especially rutin and possibly quercetin [4,54]. It has been reported that buckwheat extracts have a strong inhibitory effect on, e.g., inflammatory mediators in lipopolysaccharide-stimulated RAW 264.7-induced and peritoneal macrophages [55]. In the publication by Karki et al., 2013, buckwheat extract and rutin significantly inhibited the expression of COX-2, which may suggest their beneficial role in the treatment of inflammation [4]. Almuhayawi et al. (2021) reported that buckwheat extracts directly inhibited the activity of COX-2 and lipoxygenase [56].

Thus, *Fagopyrum esculentum* Moench and the flavonoids it contains are potential natural therapeutic agents against inflammation [23].

### 2.4. Saccharomyces cerevisiae var. boulardii

Digestion increases the antioxidant capacity of cereal and pseudocereal products. It is worth noting that digestibility is, therefore, considered to be an essential factor in enhancing the antioxidant capacity of foods such as whole grains. In the study by Kim et al., 2013, it was noticed that consumption of buckwheat simultaneously improved antioxidant processes and peroxidation, reducing the damage caused by oxidative stress [4,57,58].

There is a growing interest in adding probiotic cultures to foods. The purpose of this is to develop foods with health-promoting properties, and it is worth emphasizing that functional foods use many lactobacilli. Despite the presence of yeast in many dairy products, the active use of yeast as a probiotic has been limited. *Saccharomyces cerevisiae* var. *boulardii* is classified as a yeast species with probiotic properties. It is used prophylactically and therapeutically in the treatment of various diarrheal diseases [59,60].

*Saccharomyces cerevisiae* var. *boulardii* affects the synthesis of active phytochemicals, such as phenols, and including isoflavones [41,42]. Thus, increasing the antioxidant capacity of the products. Furthermore, when it comes to improving nutritional value, this yeast is known to catalyze the breakdown of phytate in the diet. This results in a significant improvement in the bioavailability of essential minerals [41,61,62]. The antioxidant capacity of this strain was established by Datta et al., in 2017. They compared *Saccharomyces cerevisiae* and *Saccharomyces cerevisiae* var. *boulardii* (NCYC-3264), paying attention to their antioxidant capacity, response to various stress conditions, as well as the production of important secondary metabolites. *S. boulardii* showed a better tolerance to stress but showed no significant differences in growth patterns compared to *S. cerevisiae*. Additionally, it is worth noting that *Saccharomyces cerevisiae* var. *boulardii* produced a comparatively (6 to 10 times) greater antioxidant potential, as well as 20- and 70-times the total level of flavonoids and phenols in the extracellular fraction [63]. In the publication of Fernández-Pacheco et al., 2021, twenty strains of yeast isolated from various food ecosystems, the probiotic characteristics of which had previously been studied, were presented. On the other hand, in the publication mentioned above, the yeasts were examined, among other ways, in terms of their enzymatic and antioxidant activity. All the tested yeast strains showed antioxidant activity by scavenging free radicals from the medium. It is worth mentioning that they were catalase positive, which means that the yeast has an enzyme defense system that is capable of converting reactive oxygen species from hydrogen peroxide into H_2_O and O_2_ [64].

### 2.5. Dietary Fiber

**Figure 3:**
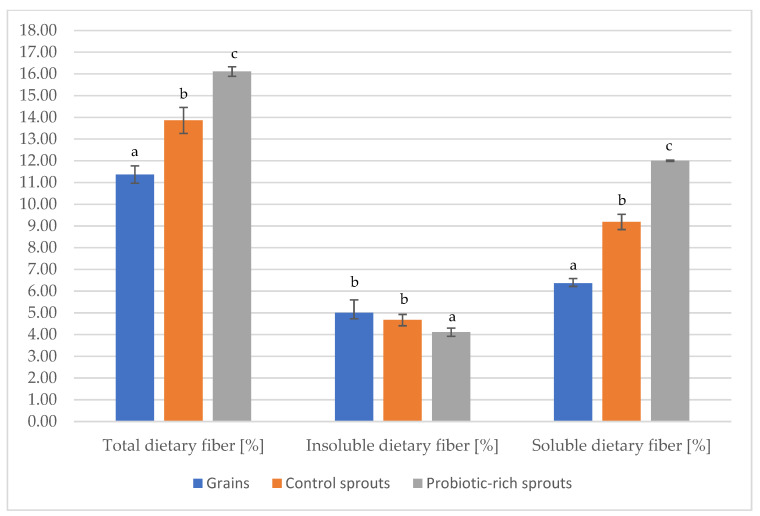
The content of total, soluble, and insoluble fiber in various forms of buckwheat. Mean values with different letters above the bars are statistically significantly different (*p* ≤ 0.05). Statistical analysis for each presented dietary fiber fraction was performed separately.

Figure 3 shows the content of total, soluble, and insoluble fiber in various forms of buckwheat. In the examined seeds and buckwheat sprouts, different amounts of dietary fiber were observed. It was found that probiotic-rich sprouts had the highest content of total dietary fiber (16.11%), while the lowest content was found in seeds (11.37%). The dominant dietary fiber fraction in probiotic-rich sprouts was soluble dietary fiber. Taking into account the level of soluble dietary fiber, the various forms of buckwheat studied can be organized as follows: probiotic-rich sprouts > control sprouts > grains. In the case of the insoluble dietary fiber fraction, probiotic rich sprouts were characterized by a statistically lower amount compared to the control sprouts and seeds.

The publication of Górecka et al., 2009, showed that buckwheat seeds have a higher content of insoluble than soluble fiber [65]. Interestingly, this is the opposite result to that obtained in the present work.

An increase in the dietary fiber in the probiotic-rich sprouts may be explained by the utilization of storage materials in growing sprouts and probiotic yeast. This caused an increase in dietary fiber in the dry biomass of the sprouts. This confirmed the results obtained in the publication by Molska et al., 2022, where the amount of resistant starch was higher in modified sprouts than in control sprouts [32]. The general behavior of resistant starch is physiologically similar to that of fermenting, soluble fiber [66]. On the other hand, β-glucan belongs to the soluble fraction of dietary fiber. Buckwheat may contain about 20 g/100 g DM of β-glucan. Moreover, yeast is also a source of this compound. The yeast cell wall (genus *Saccharomyces*) consists, inter alia, of β-glucans and mannoprotein [67,68].

The interaction between phenolic compounds and dietary fiber components affects the bioavailability, bioaccessibility, and bioactivities of phenolic compounds in foods. Studies by other authors have shown the nature of the interaction between different groups of phenolic compounds and the basic components of the cell wall matrix (e.g., polysaccharide groups in dietary fiber). Using pectin and cellulose as cell wall models, it was shown that phenolic acids and anthocyanins interact with both polysaccharides [69,70].

### 2.6. Principal Component Analysis

**Figure 4:**
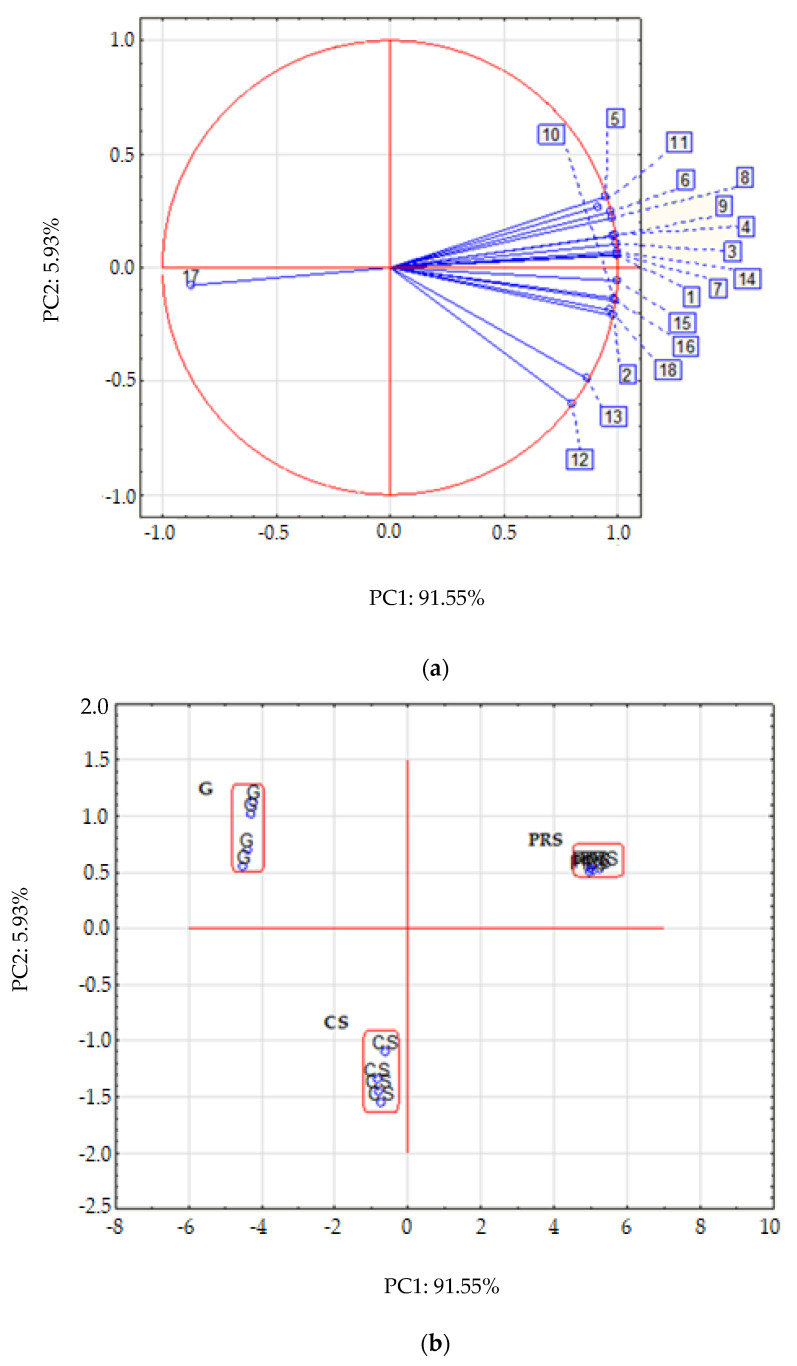
Principal components analysis (PCA) for the tested parameters (projection of variables onto the plane of factors PC1 × PC2 (**a**), projection of cases onto the plane of factors (**b**)). Abbreviations: 1. COX-1 before digestion, 2. COX-1 after digestion, 3. COX-2 before digestion, 4. COX-2 after digestion, 5. LOX before digestion, 6. LOX after digestion, 7. Total phenol compounds, 8. Total flavonoids content before digestion, 9. Total flavonoids content after digestion, 10. ABTS^+•^ before digestion, 11. ABTS^+•^ after digestion, 12. Metal chelating activity before digestion, 13. Metal chelating activity after digestion, 14. Reducing power before digestion, 15. Reducing power after digestion, 16. Soluble fiber, 17. Insoluble fiber, 18. Total dietary fiber.

For a better understanding of the interactions between the analyzed values, we conducted a principal components analysis (PCA), Figure 4a,b. The dependencies of the variables are presented in Figure 4a. The created model (a system of 2 principal components) explained 97.48% of the overall variability. The interdependence of the vectors (Figure 4a) suggested that the content of total flavonoids before and after digestion, as well as the total content of phenols, showed a negative correlation with the insoluble fiber fraction. On the other hand, the total phenolic content and the total flavonoid content before and after digestion correlated positively with each other, as well as with the antioxidant indexes. The graph of the scattering of objects in the space defined by the first two main components (Figure 4b) also provides interesting information. There are three relatively compact clusters of points, depicting individual groups. Among which, the probiotic-rich group forms the strictest cluster. A Spearman correlation analysis also confirmed the correlations shown in the PCA analysis (Table 3).

Holasova et al. (2002) noted a strong correlation between the total phenol content and the antioxidant capacity of buckwheat seeds. They indicated that the total phenolics content probably indicates the antioxidant capacity [71]. However, it is worth noting that Hung and Morita (2008) found that the free phenolic compounds had a greater share in the scavenging capacity of 2,2-diphenyl-1-picrylhydrazyl radicals, as well as the total antioxidant capacity of the buckwheat fraction, than the bound phenolic compounds [72].

## 3. Materials and Methods

All chemicals used in the analyzes were purchased from Sigma Aldrich, Poznań, Poland, unless otherwise stated.

### 3.1. Buckwheat Sprouting

*Fagopyrum esculentum* Moench seeds were purchased from PNOS S.A. in Ożarów Mazowiecki, Poland. Buckwheat seeds were disinfected in 1% (*v*/*v*) sodium hypochloride (Sigma-Aldrich. USA) for 10 min. Afterwards, grains were washed with distilled water to reach neutral pH and soaked in distilled water (CS) or in *Saccharomyces cerevisiae* var. *boulardii* water suspension (1 × 10^7^ CFU per 1 g of seeds; PRS) for 4 h. Germination was performed in a growth chamber (SANYO MLR-350H, Japan) on plates lined with absorbent paper for 3 days. Seedlings were sprayed with Milli-Q water daily. Germinated seeds (sprouts) were manually collected and rinsed with distilled water. Then, they were freeze-dried, milled, and frozen [29].

### 3.2. Phenolic Content

#### 3.2.1. Extraction Procedure

##### Solid: Solvent Extraction

Lyophilized samples of seeds and sprouts (100 mg) were mixed with 5 mL of 50% methanol. Samples were sonicated at room temperature (25 ± 1 °C) (3 intervals of 30 s; 42 kHz, 135 W; Branson Ultrasonic Corporation, Brookfield, WI, USA) and extracted for 30 min in a labor shaker (MS3 Basic, Ika, Wilmington, DE, USA, 150 rpm). Then the samples were centrifuged (20 min 6800× *g*). The resulting supernatants were kept at −20 °C prior to analysis as chemical extracts (CE).

##### In Vitro Digestion

In vitro digestion was performed as described by Minekus et al., with some modifications [73,74]. After digestion, the samples were centrifuged (15 min 6900× *g*) and the supernatants were mixed with an equal volume of methanol to stop digestion. The potentially bioavailable fractions were frozen and kept at −20 °C.

#### 3.2.2. Qualitative–Quantitative Analysis of Phenolic Compounds

The extracts for solid:solvent extraction were suspended in water (10 mL) and passed through a C18 Sep-Pak cartridge (360 mg, 55–105 µm) (Waters Associates, Milford, MA, USA) preconditioned with water. First, the cartridge was washed with water (10 mL), to remove sugars. Then, MeOH (10 mL) was used to elute the phenolic compounds. This fraction was evaporated to dryness; re-dissolved in 50% MeOH for analysis. General phenolic profiles and structural information were collected using reverse phase ultra-efficiency liquid chromatography (UPLC)-PDA-MS/MS Waters ACQUITY (Waters. Milford, MA, USA). This consisted of a binary solvent manager, sample manager, photodiode array detector (PDA), and triple quadrupole detector (TQD) operating with negative electrospray ionization.

Ion source parameters were as follows: cone voltage 35 V, capillary voltage 3 kV, extractor 3 V, RF lens 100 mV, source temperature 120 °C, desolvation temperature 350 °C, desolvation gas flow 800 L/h, cone gas flow 100 L/h, and the collision gas flow 300 µL/min. Collision cell parameters: collision energy 22 eV and input −2, output 0.5. The parameters of quadrupole 1 were set to achieve the maximum mass resolution. The LM and HM resolutions were set to 15 and the ion energy to 0.8. The collision cell parameters for the MS/MS experiments were as follows: gas collision pressure (argon), 1.5 × 10^−3^ mbar, and collision energy 15 or 30 eV. Acquisition in the MS scan mode and the product ion scan were performed in the centroid mode monitoring mode from 100 to 1200 *m*/*z*. Phenolic acids were separated on an Acquity BEH column with a diameter of 100 mm × 2.1 mm, 1.7 µm (Waters). A linear gradient of 8.5 min was applied from 80 to 100% solvent B (40% acetonitrile containing 0.1% formic acid) in solvent A (water containing 0.1% formic acid) at 0.35 mL/min. Results are expressed in µg per g dry weight (d.w.) as the equivalent of kaempferol 3-O-glucoside [75]. The method was validated for parameters such as the linearity, accuracy (relative error, RE), limit of detection (LOD), limit of quantification (LOQ), and precision (relative standard deviation, RSD). Stock standard solutions of the polyphenols were prepared with methanol. Six calibrators of each standard were prepared by dilution of stock solutions, and the calibration curve was generated by plotting the peak area ratio of the polyphenol versus the nominal concentration. A regression equation was obtained using weighted (1/c2) least-squares linear regression. The LOD was determined as a signal-to-noise ratio (S/N) of 3:1, and the LOQ was determined as a S/N of >10. An acceptable RE within ±20% and an RSD not exceeding 20% should be obtained.

#### 3.2.3. Quantitative Analysis of Total Flavonoids Content (TFC)

The total content of flavonoids was determined according to the method described by Haile and Kang [76]. A 1 mL aliquot of the test solution was mixed with 0.3 mL of NaNO_2_ (5% *w*/*v*). After 5 min, 0.5 mL of AlCl_3_ (2% *w*/*v*) was added. Standard solutions of flavonoids with a concentration of 100 µM were used. The sample was mixed and neutralized with 0.5 mL of 1 M NaOH solution 6 min later. The resulting mixture was allowed to stand for 20 min at room temperature. The absorbance at 510 nm was then measured. Total flavonoid content was calculated as quercetin equivalent (QE) in mg/g dry weight (d.w.).

### 3.3. Pro-Health Properties

#### 3.3.1. Antiradical Activity (ABTS^+•^)

Experiments were performed using an ABTS bleaching test [77]. The radical cation ABTS (ABTS^+•^) was prepared by reacting a 7mM ABTS stock solution with 2.45 mM potassium persulfate (final concentration). Leaving the mixture in the dark at room temperature for at least 6 h before use. The ABTS^+•^ solution was diluted to an absorbance of 0.7 ± 0.05 at 734 nm (Lambda 40 UV-Vis spectrophotometer, Perkin Elmer Inc. Waltham, MA, USA). Then, 50 µL of the extract obtained after in vitro digestion was added to 1.45 mL of the ABTS^+•^ solution. The absorbance was then measured at a final time of 15 min. The ability of the extracts to quench the ABTS free radical was calculated as a Trolox equivalents (TE) in mg per g of dry weight (d.w.).

#### 3.3.2. Reducing Power (RP)

The reduction power was determined using the method developed by Pulido, Bravo, and Saura-Calixto 2000 [78]. The activities were expressed as Trolox equivalents in mg/g dry weight (d.w.).

#### 3.3.3. Metal Chelating Activity (CHP)

Chelating power was determined using the method of Guo et al., 2001 [79]. Chelating power was expressed as EDTA equivalent in mg per g of dry weight (d.w.).

#### 3.3.4. Ability to Inhibit the Activity of Cyclooxygenases (COX-1 and COX-2)

The ability of the extracts obtained to inhibit cyclooxygenase-1 and cyclooxygenase-2, after chemical extraction and digestion in vitro, was determined using a COX colorimetric inhibitor screening assay kit (Cayman Chemical, No. 701050). The results were expressed in inhibitory units (UIs) per g d.w. One IU was defined as inhibition of 1U of enzyme activity in 1 min.

#### 3.3.5. Ability to Inhibit the Activity of Lipoxygenase (LOX)

A lipoxygenase inhibitory (LOXI) assay was carried out using linoleic acid as a substrate with the method described by Szymanowska et al. [80] adapted to a microplate reader. One unit of LOX activity was defined as an increase in absorbance of 0.001 per minute at 234 nm (equivalent to the oxidation of 0.12 μmole of linoleic acid). The results were expressed in inhibitory units (UIs) per g d.w. One IU was defined as inhibition of 1U of enzyme activity in 1 min.

### 3.4. Theoretical Approaches

The following factors were identified to better understand the potential bioaccesibility of biologically active extracts [75].

The gastrointestinal digestibility index (IA), which is an indicator of the bioaccesibility of antioxidants released in the digestive tract, was calculated as follows:

IA = A_GDI_/A_ce_

where A_ce_ is the activity of chemical extract (CE), and A_GDI_ is the activity of the extracts after simulated gastrointestinal digestion (GDI).

### 3.5. Dietary Fiber

Total dietary fiber (TDF), insoluble dietary fiber (IDF), and soluble dietary fiber (SDF) contents were measured using a Megazyme total dietary fiber analysis kit (Megazyme International Ireland Ltd., Wicklow, Ireland). The sum of IDF and SDF was the total dietary fiber (TDF). The content is expressed as a percentage.

### 3.6. Statistical Analysis

All experimental results were mean ± S.D. of three independent experiments (n = 9). One-way analysis of variance (ANOVA) and Turkey’s post-hoc test were used to compare groups (seeds, as well as control and elicited sprouts) (STATISTICA 6, StatSoft, Inc., Tulsa, USA). Differences were considered significant at *p* ≤ 0.05. The correlation between tested parameters was determined using principal component analysis (PCA). The Spearman correlation coefficient (*R*) and *p*-value were used to show correlations and their significance. Differences of *p* < 0.05 were considered significant.

## 4. Conclusions

The germination process changed the content of individual phenolic acids, as well as the parameters of the antioxidant activity in sprouts. When comparing the control and modified sprouts, a significant change was noticed in most phenolic acids. As a consequence, a higher antioxidant activity was noted. An association with anti-inflammatory activity and dietary fiber was also found. This is also indicated by the PCA analysis, which showed a positive correlation between total phenolic content and the total flavonoid content before and after digestion, correlating positively with each other, as well as with the antioxidant indexes. Modified buckwheat sprouts were characterized by the highest content of epicatechin gallate methyl derivative among the marked phenolic compounds.

Referring to the hypotheses in the work, the following points can be made:

1. A change in the amount of bioactive compounds was noticed in the modified buckwheat sprouts compared to the control sprouts;

2. the nutraceutical potential of the raw material was changed.

The presented research gives a new direction for the use of this raw material; common buckwheat sprouts. This information may be helpful for the food industry, aiming at producing buckwheat-based products with better nutritional and health properties.

## Figures and Tables

**Table 1:** Qualitative–quantitative analysis of phenolic compounds in buckwheat forms.

Compound		Rt	λ_max_	(M-H) m/z		Sample		
		min	nm	MS	MS/MS	Seeds (G)	Control Sprouts (CS)	Probiotic-Rich Sprouts (PRS)
*Phenolic acids*								
**1**	Caffeoyl-glucoside	2.19	288	341	179. 143	47.29 ± 0.41 ^a^	53.23 ± 0.06 ^a,b^	55.48 ± 0.52 ^b^
**2**	Caffeoyl-rhamnopyranosyl-glucopyranosyl-glucopyranoside	2.47	289	649	487. 179	24.07 ± 0.54 ^a^	27.80 ± 0.25 ^a^	29.57 ± 0.92 ^a^
**3**	Caffeoyl-rhamnopyranosyl-glucopyranosyl	3.50	288	487	179	79.80 ± 0.30 ^a^	92.04 ± 0.05 ^b^	118.23 ± 0.04 ^c^
*Flavan-3-ols*								
**1**	Unknown catechin derivate	2.57	272	535	515. 267	73.52 ± 0.01 ^c^	61.92 ± 0.23 ^b^	61.89 ± 0.08 ^a^
**2**	(Epi) afzelechin-(epi)-catechin	2.76	277	561	289. 245	9.47 ± 0.25 ^a^	38.24 ± 0.89 ^b^	56.80 ± 0.13 ^c^
**3**	Catechin-glucoside	2.91	276	451	289	39.06 ± 0.43 ^a^	62.08 ± 0.94 ^b^	113.90 ± 0.71 ^c^
**4**	Catechin-3-*O*-glucoside-6-*O*-rutinoside	3.09	278	719	451. 289	29.55 ± 0.07 ^a^	32.60 ± 0.29 ^b^	46.19 ± 0.92 ^c^
**5**	Caffeoyl-glucoside	3.31	288	341	179	2.31 ± 0.99 ^a^	17.55 ± 0.5 ^b^	33.51 ± 0.38 ^c^
**6**	(+)Catechin	3.34	276	289	-	11.59 ± 0.11 ^a^	27.85 ± 0.1 ^b^	45.14 ± 0.75 ^c^
**7**	Catechin-glucoside	3.40	279	451	289	18.35 ± 0.11 ^a^	29.78 ± 0.2 ^b^	42.66 ± 0.21 ^c^
**8**	Epicatechin-(4-8)-epicatechin	3.61	277	577	289	107.36 ± 0.19 ^a^	100.88 ± 0.05 ^a^	115.94 ± 0.62 ^b^
**9**	Epicatechin-(4-8)-epigallocatechin-gallate	3.70	279	729	577. 407. 289	15.79 ± 0.54 ^a^	34.96 ± 0.76 ^b^	57.89 ± 0.40 ^c^
**10**	Epicatechin gallate dimethyl derivative	3.81	284	469	425. 137	3.45 ± 0.42 ^a^	49.86 ± 0.48 ^b^	63.37 ± 0.93 ^c^
**11**	Epicatechin gallate	3.97	317	883 2 [M-H]^−^	441. 289	27.17 ± 0.805 ^a^	28.55 ± 0.45 ^a^	70.88 ± 0.34 ^b^
**12**	(-)Epicatechin	4.14	277	289	-	14.01 ± 0.74 ^a^	39.60 ± 0.62 ^b^	66.55 ± 0.07 ^c^
**13**	Catechin trimer	4.27	279	865	577. 289	11.26 ± 0.01 ^a^	19.80 ± 0.32 ^b^	32.73 ± 0.71 ^c^
**14**	Epicatechin gallate methyl derivative	4.44	292	455	441. 289	7.20 ± 0.86 ^a^	113.27 ± 0.02 ^b^	138.25 ± 0.98 ^c^
**15**	Epicatechin trimer	4.62	279	865	577. 289	60.67 ± 0.18 ^b^	22.61 ± 0.11 ^a^	57.14 ± 0.3 ^b^
**16**	Epiafzelechin-epicatechin-gallate dimethyl derivative	6.92	279	741	605. 469. 271	20.29 ± 0.22 ^a^	10.75 ± 0.35 ^a^	19.21 ± 0.98 ^a^
**17**	Epiafzelechin-epicatechin-gallate methyl derivative	7.88	271	727	601. 407. 289	21.39 ± 0.2 ^b^	5.68 ± 0.16 ^a^	12.32 ± 0.62 ^a^
*Flavonols*								
**1**	Orientin	4.67	269. 347	447	285	7.31 ± 0.43 ^a^	3.44 ± 0.54 ^a^	43.51 ± 0.42 ^b^
**2**	Isorientin	4.87	270. 312	447	285	ND	6.42 ± 0.56 ^a^	35.83 ± 0.25 ^b^
**3**	Quercetin-3-*O*-rutinoside	5.24	255. 352	609	301	41.28 ± 0.88 ^a^	52.48 ± 0.34 ^b^	88.37 ± 0.42 ^c^
**4**	Vitexin	5.35	269. 329	431	269	ND	20.09 ± 0.87 ^a^	121.03 ± 0.58 ^b^
**Total phenols compounds (µg/g d.w.)**						672.14 ± 0.92 ^a^	951.42 ± 1.82 ^b^	1526.34 ± 3.33 ^c^

Mean values with different letters in the row are statistically significantly different, with significance indicated as *p* ≤ 0.05. “±” means standard deviation. ND—not detected.

**Table 2:** Antioxidant activity and total flavonoid content in various forms of buckwheat.

	Before Digestion			After Digestion		
	Seeds (G)	Control Sprouts (CS)	Probiotic-Rich Sprouts (PRS)	Seeds (G)	Control Sprouts (CS)	Probiotic-Rich Sprouts (PRS)
Total flavonoids content (mg QE per g d.w.)	14.10 ± 0.64 ^a^	20.88 ± 0.59 ^b^	52.98 ± 0.77 ^c^	4.88 ± 1.18 ^a^	7.75 ± 0.78 ^b^	15.75 ± 0.62 ^c^
ABTS^+•^ (mg TE/1 g)	5.77 ± 0.83 ^a^	12.63 ± 0.50 ^b^	19.78 ± 1.37 ^c^	8.82 ± 0.42 ^a^	9.13 ± 0.54 ^a^	11.05 ± 0.29 ^b^
Metal chelating activity (mg EDTA/g d.w.)	4.08 ± 0.5 ^a^	11.58 ± 0.34 ^b^	11.70 ± 0.13 ^b^	11.40 ± 1.23 ^a^	102.15 ± 8.10 ^b^	117.78 ± 4.95 ^c^
Reducing power (mg TE/g d.w.)	11.24 ± 0.75 ^a^	17.59 ± 0.84 ^b^	31.15 ± 0.42 ^c^	2.87 ± 0.05 ^a^	6.50 ± 0.11 ^b^	11.24 ± 0.34 ^c^
COX-1 inhibitory activity (IU/g d.w.)	1664.18 ± 0.37 ^a^	2119.97 ± 4.51 ^b^	3037.15 ± 2.30 ^c^	390.20 ± 0.39 ^a^	682.00 ± 5.24 ^b^	904.00 ± 4.92 ^c^
COX-2 inhibitory activity (IU/g d.w.)	1215.42 ± 0.50 ^a^	1562.75 ± 0.50 ^b^	2431.24 ± 0.17 ^c^	128.00 ± 0.50 ^a^	213.62 ± 0.25 ^b^	470.00 ± 0.25 ^c^
LOX inhibitory activity (IU/g d.w.)	442.78 ± 5.91 ^a^	740.80 ± 10.55 ^b^	4721.56 ± 9.34 ^c^	693.94 ± 12.91 ^a^	773.69 ± 8.35 ^b^	1250.00 ± 5.52 ^c^

Mean values with different letters in the row are statistically significantly different, with significance indicated as *p* ≤ 0.05. “±” means standard deviation.

**Table 3:** Correlation between the bioactive compounds and the antioxidant and anti-inflammatory activities.

		Total Flavonoids Content (mg QE per g d.w.)		Total Phenols Compounds (µg/g DM)
		Before Digestion	After Digestion	
ABTS^+•^ (mg TE/1 g)	Before digestion	R = 0.872155 *p* < 0.00	R = 0.915499 *p* < 0.00	R = 0.947758 *p* < 0.00
	After digestion	R = 0.770579 *p* < 0.00	R = 0,721835 *p* < 0.00	R = 0.784862 *p* < 0.00
Metal chelating activity (mg EDTA/g d.w.)	Before digestion	R = 0.819616 *p* < 0.00	R = 0.713033 *p* < 0.00	R = 0.814480 *p* < 0.00
	After digestion	R = 0.853147 *p* < 0.00	R = 0,882262 *p* < 0.00	R = 0.946100 *p* < 0.00
Reducing power (mg TE/g d.w.)	Before digestion	R = 0.905269 *p* < 0.00	R = 0.945328 *p* < 0.00	R = 0.949425 *p* < 0.00
	After digestion	R = 0.888112 *p* < 0.00	R = 0.938502 *p* < 0.00	R = 0.946100 *p* < 0.00
COX-1 inhibitory activity (IU/g d.w.)	Before digestion	R = 0.882663 *p* < 0.00	R = 0.924302 *p* < 0.00	R = 0.947758 *p* < 0.00
	After digestion	R = 0.892807 *p* < 0.00	R = 0.909894 *p* < 0.00	R = 0.951101 *p* < 0.00
COX-2 inhibitory activity (IU/g d.w.)	Before digestion	R = 0.950583 *p* < 0.00	R = 0.873902 *p* < 0.00	R = 0.956183 *p* < 0.00
	After digestion	R = 0.888213 *p* < 0.00	R = 0.923267 *p* < 0.00	R = 0.961347 *p* < 0.00
LOX inhibitory activity (IU/g d.w.)	Before digestion	R = 0.937063 *p* < 0.00	R = 0.864687 *p* < 0.00	R = 0.946100 *p* < 0.00
	After digestion	R = 0.931700 *p* < 0.00	R = 0.832752 *p* < 0.00	R = 0.947758 *p* < 0.00
Total dietary fiber (%)		R = 0.904644 *p* < 0.00	R = 0,888112 *p* < 0.00	R = 0.956183 *p* < 0.00
Insoluble dietary fiber (%)		R = −0.791564 *p* < 0.00	R = −0.838378 *p* < 0.00	R = −0.836660 *p* < 0.00
Soluble dietary fiber (%)		R = 0.904644 *p* < 0.00	R = 0.888112 *p* < 0.00	R = 0.956183 *p* < 0.00

Statistically significantly different, with significance indicated as *p* < 0.05.

## Data Availability

Not applicable.
